# Antimicrobial properties of tomato juice and peptides against typhoidal *Salmonella*

**DOI:** 10.1128/spectrum.03102-23

**Published:** 2024-01-30

**Authors:** Ryan S. Kwon, Gi Young Lee, Sohyoung Lee, Jeongmin Song

**Affiliations:** 1 Department of Microbiology and Immunology, Cornell University, Ithaca, New York, USA; US Department of Agriculture, Washington, DC, USA

**Keywords:** *Salmonella*, antimicrobials, antimicrobial peptides, tomato, Gram-negative bacteria, enteric pathogens

## Abstract

Tomatoes are readily available and affordable vegetables that offer a range of health benefits due to their bioactive molecules, such as antioxidants and antimicrobials. In contrast to the widely recognized antioxidant properties of tomatoes, their antimicrobial properties remain largely unexplored. Here, we present our findings on the antimicrobial properties of tomato juice and peptides, namely, tomato-derived antimicrobial peptides (tdAMPs), in relation to their effectiveness against typhoidal *Salmonella*. Our research has revealed that tomato juice demonstrates significant antimicrobial properties against *Salmonella* Typhi, a pathogen that specifically affects humans and is responsible for causing typhoid fever. By employing computational analysis of the tomato genome sequence, conducting molecular dynamics simulation, and performing functional analyses, we have successfully identified two tdAMPs, namely, tdAMP-1 and tdAMP-2. These tdAMPs have demonstrated potent antimicrobial properties by effectively disrupting bacterial membranes. The efficacy of tdAMP-2 is shown to be more effective than tdAMP-1. The efficacy of tdAMP-1 and tdAMP-2 has been demonstrated against drug-resistant *S*. Typhi, as well as hyper-capsular *S*. Typhi variants that possess hypervirulent characteristics, which are presently circulating in countries with endemicity. Tomato juice, along with the two tdAMPs, has demonstrated effectiveness against uropathogenic *Escherichia coli* as well. This underscores their potential as viable agents in combating certain Gram-negative pathogens. This study provides valuable insights into the development of effective and sustainable public health strategies that utilize tomato and its derivatives as lifestyle interventions.

**IMPORTANCE:**

In this study, we investigate the antimicrobial properties of tomato juice, the most widely consumed affordable vegetables, as well as tomato-derived antimicrobial peptides, in relation to their effectiveness against foodborne pathogens with an emphasis on *Salmonella* Typhi, a deadly human-specific pathogen.

## INTRODUCTION

Tomatoes, scientifically known as *Solanum lycopersicum*, are highly popular vegetables globally, owing to their exceptional taste and wide range of applications in various culinary traditions. The nutritional composition of tomatoes, including their abundance of beneficial compounds like lycopene, polyphenols, and vitamins, has been extensively studied and linked to a wide range of health advantages (1–6). Additionally, tomatoes have the advantage of being able to thrive in a wide range of regions with varying climate conditions. According to the 2021 report by the Food and Agriculture Organization of the United Nations (faostat@fao.org), Asia is the leading global producer of tomatoes, representing 64.8% of the total global tomato production. Europe, Africa, North America, and South America account for 13.3%, 11.6%, 5.98%, and 3.95% of the total tomato production, respectively.

Considerable research has been dedicated to investigating the antioxidant properties of various substances. In contrast, a limited number of studies have observed that peptides obtained from extracts of seeds, leaves, stems, and peels exhibit antimicrobial activity against foodborne pathogens (7–9). Antimicrobial peptides (AMPs) are naturally occurring compounds also found in plants that play a crucial role in the innate immune system’s ability to combat infections within their environment (10–12). It has been found that tomatoes possess AMPs, which are recognized for their strong and wide-ranging activities (13–16). The AMPs are cationic, amphipathic peptides that are found in animals and plants. They exhibit rapid antimicrobial activity by directly targeting the cell membrane (16, 17). The amphipathicity of AMPs allows for their interaction with the negatively charged head groups of the lipid bilayer in the membrane. This interaction is followed by their penetration into the membrane, resulting in the formation of pores. These pores ultimately lead to membrane permeabilization and subsequent bacterial death (18, 19). In addition to their direct antimicrobial properties, AMPs have the ability to modulate immune responses by facilitating the recruitment and activation of immune cells, as well as stimulating the release of cytokines and chemokines (19).

The global prevalence of various enteric pathogens poses a substantial risk to our collective well-being. *Salmonella enterica* serovar Typhi (*S*. Typhi), the causative agent of typhoid fever, which is a human-specific pathogen, has been widely acknowledged as the primary cause of mortality resulting from enteric pathogen infections (20, 21). Every year, there are 21 million reported cases of typhoid fever, resulting in 200,000 fatalities globally. Despite the availability of typhoid vaccines, numerous developing countries continue to face substantial obstacles in accessing these vital resources. Additionally, the issue of antibiotic resistance poses a significant risk, particularly among malnourished children (22–27).

In a study conducted by Allen et al. (28), it was found that a significant proportion of children (aged 6–23 months) residing in 64 developing countries, specifically 45.7%, do not include vegetables or fruits in their diet, indicating a prevalence of malnutrition in this population. In Pakistan, there has been a significant emergence and global spread of extensively drug-resistant *S*. Typhi, particularly in recent years. It is worth noting that in 2018, approximately 65% of children in the country did not consume an adequate amount of vegetables or fruits, which was the highest across Asian countries, with India following closely (28). This correlation may be attributed to the association between the consumption of fruits and vegetables and the enhancement of immunity and antibacterial activity (26–28).

Given that the tomato fruit is more frequently consumed by individuals compared to other parts of the plant where antimicrobial activities have been reported (7–9), it would be more practical to assess the antimicrobial properties of tomato juice as a potential approach for lifestyle interventions. In this study, we investigated the antimicrobial properties of tomato juice, the most widely consumed affordable vegetables, as well as tomato-derived antimicrobial peptides (tdAMPs), in relation to their effectiveness against foodborne pathogens with an emphasis on *S*. Typhi.

## RESULTS

### Tomato juice exhibits significant antimicrobial activity against *S*. Typhi

**Fig 1:**
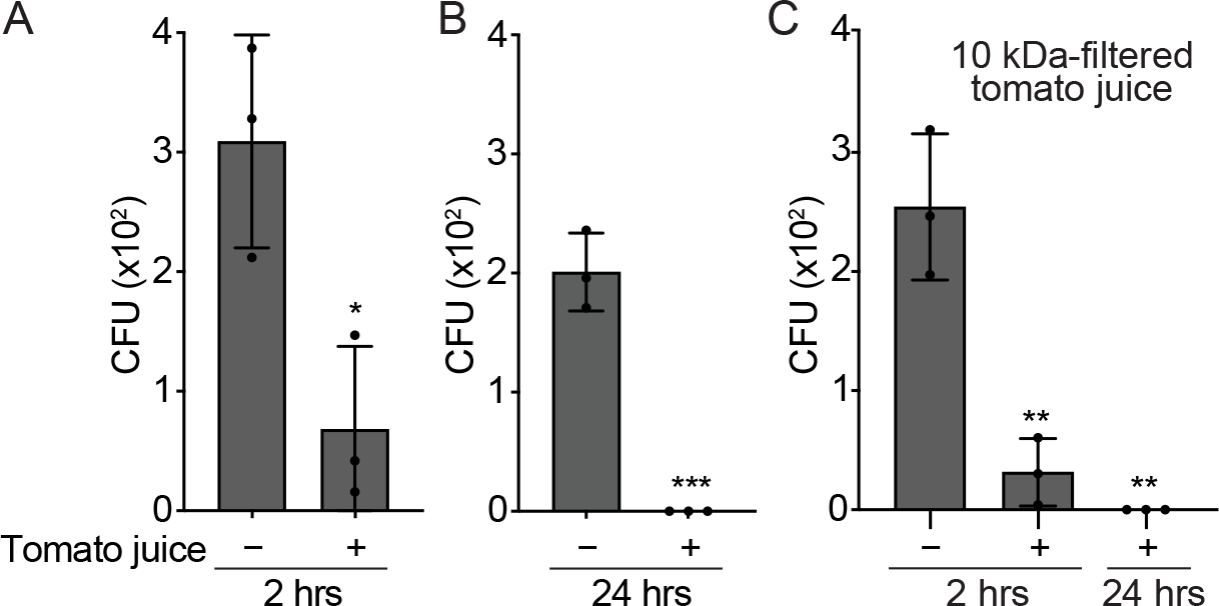
Tomato juice exhibits significant antimicrobial activity against *S.* Typhi. (A–C) CFU assays of *S*. Typhi incubated with tomato juice at 2 (**A**) and 24 hours (**B**), or S. Typhi incubated with tomato juice that had been filtered using a 10-kDa filter, also for 2 and 24 hours (**C**). Data represent mean  ±  standard deviation of three independent experiments. Two-tailed Student *t*-tests between mock-treated and tomato juice-incubated *S*. Typhi were performed (*P < 0.05, **P < 0.01, ***P <  0.001). See also Fig. S1.

To evaluate the antimicrobial properties of the tomato fruit, we proceeded by grinding fresh tomatoes to produce tomato juice. Subsequently, we conducted an incubation experiment wherein *S*. Typhi was exposed to tomato juice. A notable reduction in the colony-forming units (CFUs) of *S*. Typhi was observed at the 2-hour mark following incubation, in comparison to the control group treated with phosphate-buffered saline (PBS) (Fig. 1A). In addition, it was observed that *S*. Typhi was effectively eliminated within a 24-hour incubation period (Fig. 1B), providing evidence of the antimicrobial properties exhibited by tomato juice. The pH of the tomato juice used in the study was determined to be approximately 4.5. To rule out the possibility that an acidic environment plays a role in *S*. Typhi growth inhibition, we carried out comparative growth tests in media with pH levels of 4.5 and 7.4. We found that the growth of *S*. Typhi was similar under both pH conditions (Fig. S1), indicating that the observed antimicrobial properties of tomato juice on *S*. Typhi bacteria are unlikely due to its acidic pH.

To determine the bioactive molecules responsible for the antimicrobial activity, the tomato juice underwent filtration using a column with a 10-kDa cutoff. We observed that the antimicrobial activity of the pass-through fraction was comparable to that of the total tomato juice, as shown in Fig. 1C. This suggests that the observed antimicrobial activity can be attributed to bioactive molecules that are smaller than 10 kDa.

### Tomato-derived AMPs exhibit significant antimicrobial activity against *S*. Typhi

**Fig 2:**
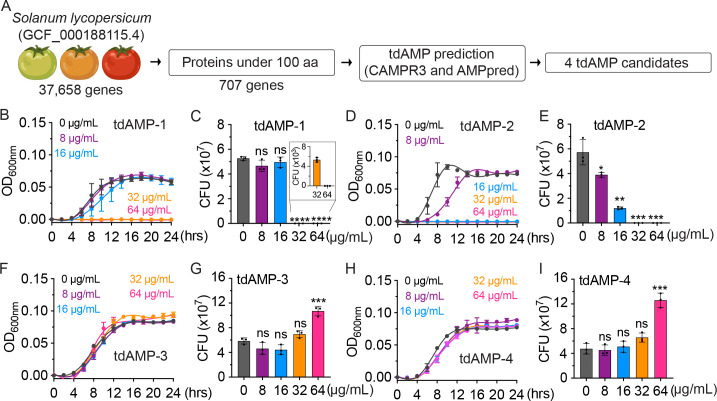
Tomato-derived AMPs exhibit significant antimicrobial activity against *S*. Typhi. (A), Our data analysis pipeline depicting selection of tdAMP candidates under 100 amino acids (aa). from *Solanum lycopersicum*’s whole-genome sequence.** (B–I) **Growth curves of *S*. Typhi treated with tdAMP-1 (**B**), tdAMP-2 (**D**), tdAMP-3 (**F**), or tdAMP-4 (**H**) at the indicated concentrations. Minimum inhibitory concentration (MIC) was determined where no growth was found after 24 hours. CFU assays of *S*. Typhi incubated for 24 hours with tdAMP-1 (**C**), tdAMP-2 (**E**), tdAMP-3 (**G**), or tdAMP-4 (**I**). Data represent mean  ±  SD of three independent experiments. Two-tailed Student *t*-tests between mock- and tdAMP-treated *S*. Typhi were performed (ns, not significant, *P < 0.05, **P < 0.01, ***P < 0.001, ****P <  0.0001). See also Fig. S2 and Table S1. Created with BioRender.

To identify potential candidates for antimicrobial peptides, a novel unbiased analysis was conducted on the complete genome sequence of *Solanum lycopersicum*. Out of the total of 37,658 genes that were examined, our attention was directed toward 707 genes that encode proteins with a length of less than 100 amino acids. This analysis was conducted using two AMP prediction tools, namely, CAMPR3 (29) and AMPpred (30). We have selected a group of candidates from AMPpred whose antibacterial probability exceeds 0.9, with a maximum probability of 1. This selection process has yielded the top 20 candidates, as shown in Fig. 2A and Table S1. After careful evaluation of the candidates, we have identified the two most promising candidates (YP_008563122.1 and XP_025885552.1) that have tested positive for both CAMPR3 and AMPpred. These candidates have been designated as tdAMP-1 and tdAMP-2, respectively. Furthermore, we have identified a gene consisting of five isotypes among the top 20 candidates. These isotypes possess a neutral isoelectric point (pI), which is in contrast to the typical basic pI characteristic observed in canonical AMPs. Therefore, we proceeded to select two out of the five isotypes of the gene, which we have designated as tdAMP-3 (XP_004247438.1) and tdAMP-4 (XP_004247441.1) (Table 1; Table S1).

**TABLE 1:** Summary of tdAMPs used in this study

tdAMP	Sequence	Length	M.W.	pI
tdAMP-1	MKIRASVRKICEKCRLIRRRGRIIVICSNPRHKQRQG	37	4.5 kDa	12.4
tdAMP-2	MMKGKNEANLKSKKRRICSGKLGRFLKEQRGRLYIVRRCVVMLLCWHD	48	5.8 kDa	10.6
tdAMP-3	MSDEEVVDPKATLEVSCKPKCVRQLKEYQACTKRIEGDESGHKHCTGQYFDYWHCIDKCVAAKLFDHLK	69	8 kDa	6.5
tdAMP-4	MSDEEVVDPKATMEVSCKPKCVRQLKDYQACTRRIEGDESGSKHCTGQYFDYWQCIDKCVAPKLFEKLK	69	8 kDa	6.8

To assess the effectiveness of the four AMP candidates (tdAMP-1, tdAMP-2, tdAMP-3, and tdAMP-4), we conducted an evaluation of their minimal inhibitory concentrations (MICs) and minimal bactericidal concentrations (MBCs) against *S*. Typhi. Both tdAMP-1 and tdAMP-2 demonstrated significant growth inhibition and killing of *S*. Typhi. Notably, tdAMP-2 (MIC = 16 µg/mL and MBC = 32 µg/mL) exhibited twice the effectiveness of tdAMP-1 (MIC = 32 µg/mL and MBC = 64 µg/mL) (Fig. 2B through E; Table 2). The determined MIC and MBC values of Melittin, used as a positive control AMP that is found in vee venom, were 16 and 64 µg/mL, respectively (Table 2). In contrast, tdAMP-3 and tdAMP-4 did not demonstrate any detectable growth inhibitory or bactericidal effects on *S*. Typhi at all tested concentrations (Fig. 2F through I). Moreover, we observed that the growth of *S*. Typhi was significantly enhanced in the presence of 64 µg/mL of tdAMP-3 or tdAMP-4 (Fig. 2G and I), which implies that *S*. Typhi might have the ability to utilize these peptides as a nutritional source for their growth. These results collectively indicate that tdAMP-1 and tdAMP-2 are effective antimicrobial peptides, whereas tdAMP-3 and tdAMP-4 are not.

**TABLE 2:** Minimum inhibitory concentration (MIC) and minimum bactericidal concentration (MBC) of tdAMP-1 and tdAMP-2 against the indicated strain^*a*^

Strain	Antibiotics	MIC (µg/mL)	MBC (µg/mL)
*S.* Typhi	tdAMP-1	32	64
	tdAMP-2	16	32
	tdAMP-3	ND	ND
	tdAMP-4	ND	ND
	Ciprofloxacin	0.004	0.016
	Melittin	16	64
*S.* Typhi gyrA S83F	tdAMP-1	32	64
	tdAMP-2	16	32
	Ciprofloxacin	0.125	0.5
Acapsular *S.* Typhi (∆tviBC)	tdAMP-1	32	64
	tdAMP-2	16	32
Hypercapsular *S.* Typhi (tviE P263S)	tdAMP-1	32–64	64
	tdAMP-2	32	32
*S.* Typhimurium LT2	tdAMP-1	32–64	64
	tdAMP-2	32–64	64
Uropathogenic *E. coli* CI5	tdAMP-1	64	64
	tdAMP-2	32–64	32–64
Uropathogenic *E. coli* J96	tdAMP-1	64	64
	tdAMP-2	32	64

^*a*^ Note that N-minimal medium was used in this study. ND, not determined.

All four tdAMPs were chemically produced and purified using high-performance liquid chromatography (HPLC), including tdAMP-1 and tdAMP-2. Given that tdAMP3 and tdAMP4 did not exhibit antimicrobial activity, it seems doubtful that the remaining trifluoroacetate (TFA) salt was the primary cause of tdAMP-1 and tdAMP-2’s antibacterial properties. To ensure that the observed antibacterial properties of tdAMP-1 and tdAMP-2 are not attributable to residual TFA salt, we dialyzed these peptides with 20 mM of acetate for 24 hours using a 1-kDa dialysis membrane and tested them for antimicrobial activity. The antibacterial activities of dialyzed tdAMP-1 and tdAMP-2 against *S*. Typhi were similar (Fig. S2A through D). The final acetate concentration ranged from 0.25 mM (for 8 µg/mL of tdAMPs) to 2 mM (for 64 µg/mL of tdAMPs). We also confirmed that these acetate concentrations have no influence on *S*. Typhi growth (Fig. S2E).

### tdAMP-1 and tdAMP-2 are effective against drug-resistant, acapsular, or hyper-capsular/hypervirulent *S.* Typhi variants

**Fig 3:**
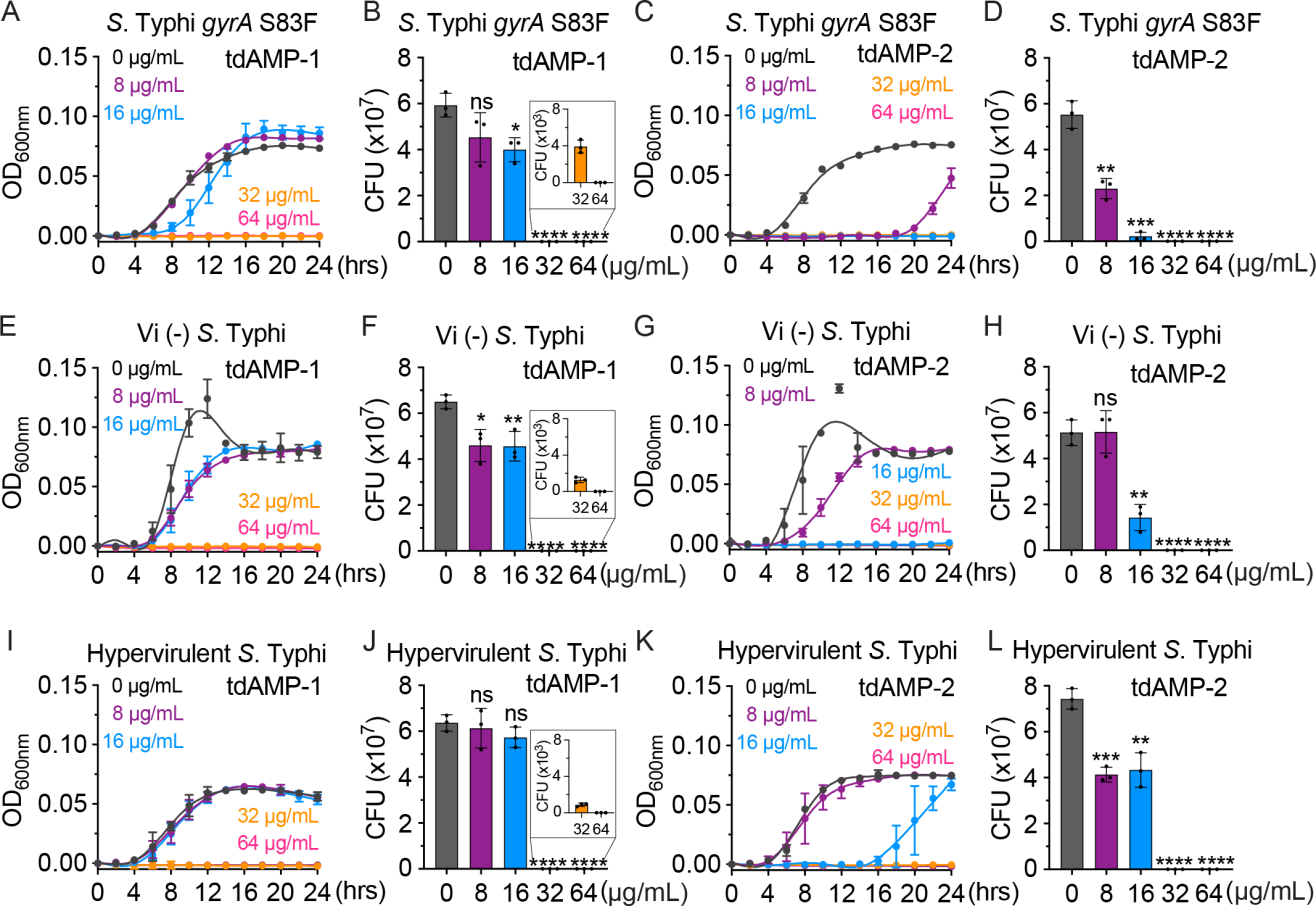
tdAMP-1 and tdAMP-2 are effective against drug-resistant, acapsular, or hyper-capsular/hypervirulent *S.* Typhi variants. (A–L) Growth curves and CFU assay results of drug-resistant *S*. Typhi (**A–D**), acapsular *S*. Typhi (**E–H**), and hyper-capsular/hypervirulent *S*. Typhi (**I–L**) treated with tdAMP-1 or tdAMP-2 at the indicated concentrations. Data represent mean  ±  SD of three independent experiments. Two-tailed Student *t*-tests between mock- and tdAMP-treated *S*. Typhi were performed (ns, not significant, *P < 0.05, **P < 0.01, ***P < 0.001, ****P <  0.0001).

Ciprofloxacin is the primary antibiotic used for the treatment of typhoid fever in regions where the prevalence of the disease is high. Nevertheless, there have been reports of resistance to it, and a frequently observed clinical mutation that leads to ciprofloxacin resistance is *gyrA* S83F (31). To evaluate the efficacy of tdAMP-1 and tdAMP-2 against drug-resistant *S*. Typhi, we employed *S*. Typhi strains carrying *gyrA* S83F mutation. The presence of the *S*. Typhi *gyrA* S83F mutation was confirmed through sequencing. It was consistently observed that there was a 32-fold increase in ciprofloxacin resistance (Table 2). Based on our investigation using the drug-resistant strain, it has been determined that both tdAMP-1 and tdAMP-2 exhibit equal efficacy against *S*. Typhi *gyrA* S83F and WT *S*. Typhi (Fig. 3A through D; Table 2).

We next explored whether tdAMP-1 and tdAMP-2 are also effective against capsular variants. Many bacterial pathogens possess capsular polysaccharides as a crucial virulence determinant, providing them with resistance against the host’s innate immune system, antibiotics, and AMPs (32). These capsular bacteria leverage this advantage to ensure their survival in the host, and this phenomenon has been associated with hypervirulence (33). To assess the efficacy of tdAMP-1 and tdAMP-2 against hyper-capsular bacteria, we have selected hyper-capsular/hypervirulent *S*. Typhi strains that are currently prevalent in endemic regions (34). Both tdAMP-1 and tdAMP-2 demonstrated efficacy against acapsular (Fig. 3E through H) and hyper-capsular/hypervirulent *S*. Typhi strains (Fig. 3I through L; Table 2). These results indicate that the tdAMPs exhibit bactericidal activity against *S*. Typhi, irrespective of the presence or absence of the Vi capsule.

### Tomato juice, tdAMP-1, and tdAMP-2 induce membrane permeabilization

To investigate the potential mechanism underlying the antimicrobial activity of tomato juice and tdAMPs, we conducted a study on membrane permeabilization. To obtain a deeper understanding of the interaction between tdAMPs and bacterial membranes, as well as to analyze the molecular-level binding affinity disparity between tdAMP-1 and tdAMP-2, we utilized molecular dynamics. This computational technique allows for the simulation of AMPs' interaction with bacterial membranes.

**Fig 4:**
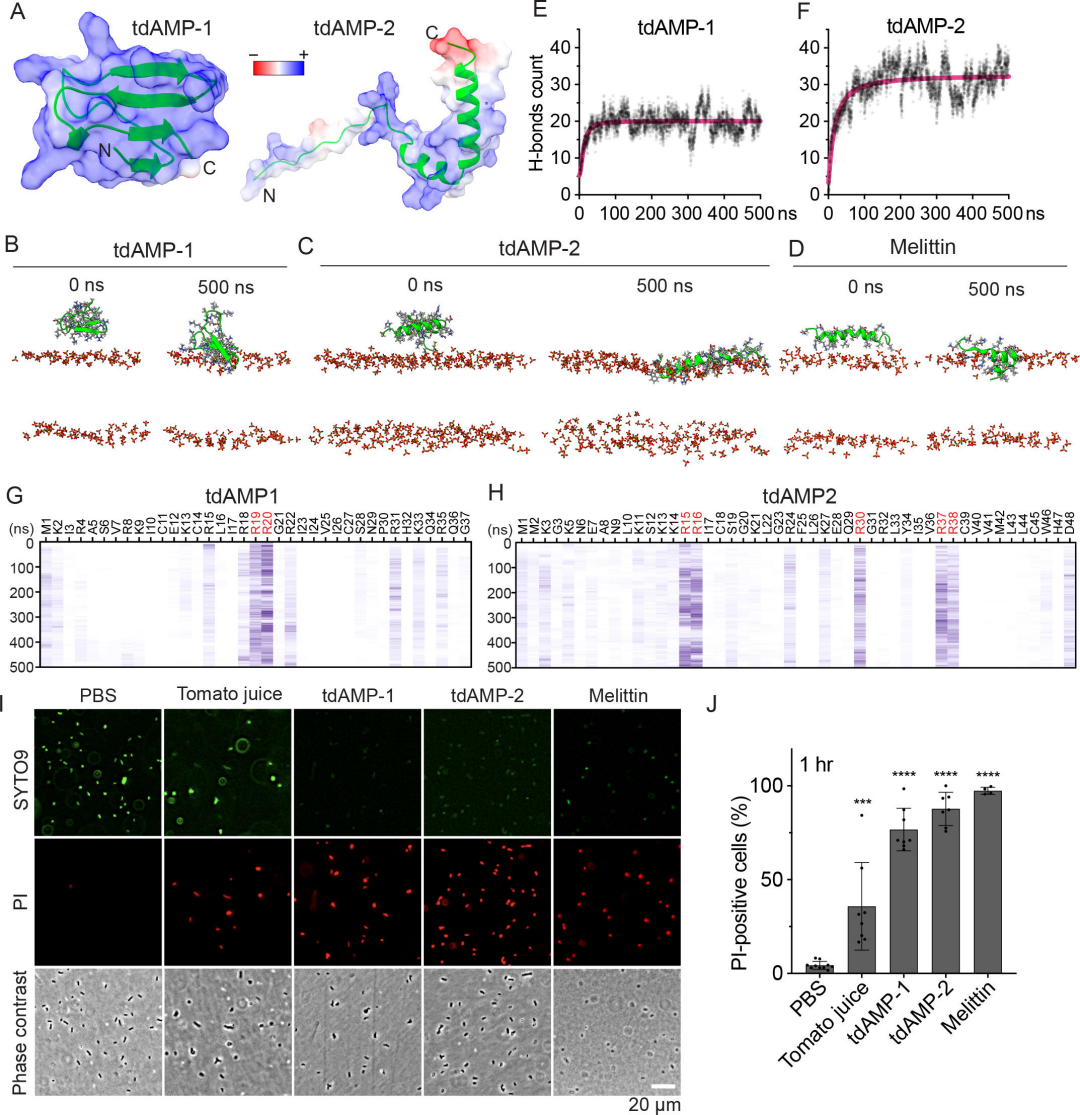
Tomato juice, tdAMP-1, and tdAMP-2 induce membrane permeabilization. (A–C) Molecular dynamics simulation of tdAMP-1 and tdAMP-2. The structures of tdAMP-1 and tdAMP-2 were predicted by AlphaFold v2 (**A**). Interactions of tdAMP-1 (**B**), tdAMP-2 (**C**), or Melittin (**D**) with the model membrane-mimicking bacterial membrane were shown. Green, backbone of tdAMP-1, tdAMP-2, and Melittin; red, phosphate group of lipids. (**E–F) **Counts of hydrogen bonds formed between the indicated tdAMP and the model membrane. (**G–H)** Heatmaps of hydrogen bonds formed between each amino acid of the indicated tdAMP (X-axis) and the model membrane by time (Y-axis). (**I–J) **Tomato juice, tdAMP-1, or tdAMP-2 induced membrane permeabilization evaluated by bacterial live/dead staining. SYTO9 (green) represents live bacteria, while PI (propidium iodide, red) represents dead bacteria.** (I) **Representative fluorescence microscopy images of bacterial cells. Ten microliters of *S*. Typhi (10^6^ CFU of bacteria) was added to 90 µL of tomato juice, or 90 µL of PBS containing 64 µg/mL of tdAMPs or 64 µg/mL of Melittin. After incubating at 37°C for 45 minutes, the bacteria were stained and analyzed. Scale bars, 20 µm. (**J) **Quantification results of three independent experiments associated with I. Data represent mean ± SD of three independent experiments. Two-tailed Student *t*-tests between PBS and tomato juice, PBS and tdAMP-treated, or PBS and Melittin-treated *S*. Typhi were performed (***P < 0.001, ****P <  0.0001).

To prepare for the simulations, we initially made predictions on the structures of tdAMP-1 and tdAMP-2 using AlphaFold v2 (35). These predicted structures were then employed as inputs for the molecular dynamics (MD) simulations, as shown in Fig. 4A. The tdAMP-1, tdAMP-2, or Melittin was applied to the model membrane, which mimics a bacterial membrane. A simulation was conducted for a duration of 500 ns, with three iterations. The tdAMP-1, tdAMP-2, and Melittin exhibited stable binding to the model membrane over a duration of 500 ns (Fig. 4B through D). Consequently, we proceeded to evaluate the strength of interaction between the tdAMPs and the model membrane by analyzing the hydrogen bonds formed between them. The tdAMP-2 demonstrated a greater than 1.5-fold increase in affinity for the bacterial membrane in comparison to tdAMP-1 (Fig. 4E and F), thereby providing support for the higher antimicrobial activity of tdAMP-2 over tdAMP-1 (Fig. 2 and 3). The residues R19/R20 were found to be crucial for the stable interaction in the case of tdAMP-1, while R15/R16/R30/R37/R38 played a significant role in the stable interaction of tdAMP-2 (Fig. 4G and H). Consistent with the findings obtained from the MD simulations (Fig. 4A through H), we observed that tdAMP-1, tdAMP-2, and tomato juice caused membrane permeabilization following a 45-minute incubation period (Fig. 4I and J). As propidium iodide (PI) stains bacteria when both their outer and inner membranes are damaged, the results indicate that tomato juice and tdAMPs possess bactericidal activity through the disruption of bacterial membranes.

### tdAMP-1, tdAMP-2, and tomato juice exhibit antimicrobial properties against various enteric pathogens

**Fig 5:**
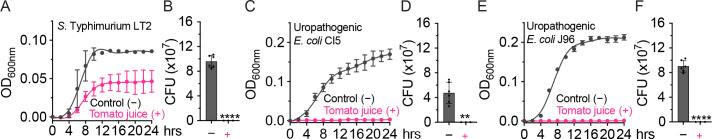
tdAMP-1, tdAMP-2, and tomato juice exhibit antimicrobial properties against various enteric pathogens. (A–F) Growth curves and CFU assay results of *S*. Typhimurium (**A–B**), uropathogenic *E. coli* CI5 (**C–D**), and uropathogenic *E. coli* J96 (**E–F**) incubated with tomato juice. Data represent mean ± SD of two to three independent experiments.

To assess the suitability of tomato juice and tdAMPs for addressing other enteric pathogens, we conducted tests on various strains including *S*. Typhimurium and two uropathogenic *E. coli* (UPEC) strains (*E. coli* CI5 and *E. coli* J96) (Fig. 5). *S*. Typhimurium is a prominent strain of non-typhoidal *Salmonella* that is known to cause acute gastroenteritis, but it is typically not considered to be life threatening for individuals (36). UPEC is recognized as the causative agent of urinary tract infections (UTIs), a condition that can result in medical complications if not promptly addressed (37–39). UTIs are among the common infectious diseases in developed countries. We found that tomato juice inhibited the growth of *S*. Typhimurium and UPEC strains (Fig. 5). After 24 hours of incubation, none of the tested strains were recovered (Fig. 5B, D, and F). Similarly, we found that the peptides, tdAMP-1 and tdAMP-2, were effective against *S*. Typhimurium, *E. coli* CI5, and *E. coli* J96 in MIC and MBC testing (Table 2). The findings collectively suggest that both tdAMPs and tomato juice exhibit effectiveness against typhoidal/non-typhoidal *Salmonella* and UPEC strains.

## DISCUSSION

In this study, we have identified antimicrobial properties in tomato juice, a widely consumed vegetable known for its popularity and affordability globally. Our research specifically focused on its effectiveness against *S*. Typhi, *S*. Typhimurium, and uropathogenic *E. coli*, as illustrated in Fig. 1 and 5. Furthermore, through computational and functional analyses, our study has identified two tomato-derived antimicrobial peptides tdAMP1 and tdAMP2 that demonstrate antimicrobial properties against a range of enteric pathogens, including typhoidal *Salmonella* (*S*. Typhi), non-typhoidal *Salmonella* (*S*. Typhimurium), and uropathogenic *E. coli* strains (Fig. 2; Table 2).

Drug-resistant enteric bacteria, including *S*. Typhi, have been observed to spread (40–43). We tested the efficacy of tdAMP-1 and tdAMP-2 against one of the most common forms of drug-resistant *S*. Typhi in this investigation. Based on our findings with *S*. Typhi bearing *gyrA* S83F, we discovered that tdAMP-1 and tdAMP-2 have equivalent efficacy against drug-susceptible and drug-resistant *S*. Typhi (Fig. 3; Table 2), emphasizing their significance and potential applicability.

Many bacterial pathogens possess capsular polysaccharides as a critical virulence factor, enabling them to evade the host’s innate immune system, antibiotics, and AMPs (32). Multiple studies have been conducted on capsulated bacterial pathogens, such as *Klebsiella pneumoniae* and *Acinetobacter baumannii*, which have provided evidence of the presence of capsular variants (33, 44–49). Intriguingly, we have observed similar adaptations in *S*. Typhi (34). These findings suggest a parallel evolution of capsulated pathogens. Therefore, we conducted an investigation to determine the efficacy of tdAMP-1 and tdAMP-2 against capsular variants. For the purpose of this investigation, we have chosen hyper-capsular/hypervirulent strains of *S*. Typhi that are currently prevalent in endemic regions (34). We found that both tdAMP-1 and tdAMP-2 demonstrated antimicrobial properties against acapsular and hyper-capsular/hypervirulent S. Typhi strains (Fig. 3; Table 2), underscoring their importance and potential utility against capsule variants.

The MD simulation is a computational technique that allows for the real-time analysis of atomic movements. It has emerged as a valuable tool for studying the atomic-level interactions between AMPs and model membranes. In our study, we conducted MD simulations to investigate the binding affinity of tdAMP-1 and tdAMP-2 with a bacterial model membrane. Our findings indicate that there is a significant difference in binding affinity between tdAMP-1 and tdAMP-2, with tdAMP-2 exhibiting a greater than 1.5-fold higher affinity for bacterial membranes than that of tdAMP-1 (Fig. 4A through H). This difference in binding affinity is in agreement with the variance in membrane permeabilization and antimicrobial activity (Fig. 4I and J; Fig. 2), which is widely recognized as a characteristic feature of AMPs acting on bacterial membranes (50–53).

Based on the fact that only four tdAMP candidates were screened with two tdAMPs with characterizations, it is reasonable to speculate that there may be many more tdAMPs present in tomato fruit. Conducting additional research will provide a comprehensive understanding of the antimicrobial properties of tomatoes, thereby making a valuable contribution to enhancing public health. It is worth emphasizing that this type of research provides valuable information not just because of incomplete or imperfect annotations of the whole-genome sequence of tomato but also because existing annotations are for their expected roles within plants rather than their impacts in humans.

In summary, this study offers valuable insights into the potential utilization of tomatoes as a natural antimicrobial food source for the promotion of public health. Given their widespread popularity and cost effectiveness, tomatoes possess considerable potential as alternative antimicrobial agents within lifestyle interventions.

## MATERIALS AND METHODS

### Prediction and synthesis of tdAMPs

The whole-genome sequence of *Solanum lycopersicum* (GCF_000188115.4) was used to collect the genes under 100 amino acids. The resulting 707 genes were analyzed with IPC (54) for calculating molecular weight and pI, CAMPR3 (29) and AMPpred (30) for AMP prediction. All tdAMP candidates (tdAMP-1, 2, 3, and 4) were chemically synthesized by GL Biochem and purified by high-performance liquid chromatography. When indicated, two tdAMPs that showed antimicrobial activity, tdAMP-1 and tdAMP-2 (0.64 mg/mL), were dialyzed with 20 mM of acetate for 24 h using a 1-kDa dialysis membrane (Spectra/Por®6 Dialysis Membrane, Spectrum Labs, cat#: 132636T). The antibacterial activity of the resultant tdAMPs was tested. In the bacterial cultures used for MIC and MBC testing, the final acetate concentration ranged from 0.25 mM (for 8 µg/mL of tdAMPs) to 2 mM (for 64 µg/mL of tdAMPs).

### Tomato juice preparation

Heirloom and Campari tomatoes, which are between mature green and breaker (tomatoes starting to change colors), were ground with a homogenizer and filtered with two sterilized 100-µm nylon mesh layers. The filtrate was centrifuged for 30 min at 20,000 × *g* at 4°C to remove residual pulps and ground cells and stored at −80°C until use.

### Bacteria strains

*S*. Typhi ISP2825 (SB2201) (55), *S*. Typhi Δ*tviBC* (SB2203), hyper-capsular *S*. Typhi (JS0047), *S*. Typhi *gyrA* S83F (designated as JS0062), *S*. Typhimurium LT2, *E. coli* CI5, and *E. coli* J96 were used in this study. Bacteria were grown overnight in 2 mL of Luria–Bertani (LB) medium at 37°C prior to use. JS0062 was constructed as previously described with minor modifications (56, 57). Briefly, the suicide vector pSB890 was digested with BamHI-HF (NEB, cat# R3136S) and NotI-HF (NEB, cat# R3189S). Inserts were amplified by PCR reactions using Herculase II Fusion DNA Polymerase (Invitrogen, cat# 600679) with primers (F1: 5′-taaaaagccccaccgcggtggcggcccgggtatacacgggaggtattgattttccag-3′, R1: 5′-ggtgtcatacactgcgaaatcgccgtggggatg-3′, F2: 5′-catccccacggcgatttcgcagtgtatgacacc-3′, R2: 5′-gtaagtgaactgcagcccgggggatcgcgcgaatgtacactttgccacgac-3′). The digested vector and two inserts were Gibson assembled (T5 exonuclease, NEB cat# M0363S; Phusion polymerase, NEB cat# M0530S; Taq DNA ligase, NEB cat# M0208L) to generate pSB890 *gyrA* S83F plasmid (designated as pJS0296). pJS0296 was transformed into *E. coli* ß2163 ∆nic35 for conjugation with *S*. Typhi and subsequently underwent homologous recombination. The strain was verified by Sanger sequencing through the Cornell Institute Biotechnology Resource Center Genomics Facility.

### Minimum inhibitory concentration and minimum bactericidal concentration determination

The MIC and MBC assays were conducted according to the CLSI guidelines (https://clsi.org), with minor modifications made to accommodate the specific conditions for the bacterial strains utilized in this study, including the use of tomato juice and acidic culture conditions. Each bacterial culture (100 µL) was added to 2 mL of PBS to measure the optical density (OD_600nm_). Each bacterial sample was further diluted to 1 × 10^5^ CFU of bacteria in 10 µL. Twofold serial diluted tdAMP in a range from 0 to 64 µg/mL in 90 µL of N-minimal medium [5 mM of KCl, 7.5 mM of (NH_4_)_2_SO_4_, 0.5 mM of K_2_SO_4_, 1 mM of KH_2_PO_4_, 10 µM of MgCl_2_, 38 mM of glycerol, 0.2% glucose, 0.1% casamino acids, and 50 mM of Tris/MES (2-(N-morpholino)ethanesulfonic acid) pH 7.4] or tomato juice was added into a 96-well plate, respectively. When *S*. Typhi was cultured, 0.005% tryptophan was also added to the media. Ten microliters (1 × 10^5^ CFU of bacteria in 10 µL) was added into the well containing tdAMP or tomato juice. Empty wells were filled with 100 µL of MilliQ water to prevent dehydration. OD_600nm_ was measured at 37°C with 30-min intervals for 24 hours. The MBC was determined by plating serially diluted samples that were harvested 24 hours after incubation. The MBC value was determined in instances where no bacteria were detected, as observed in all three independent experiments. The colonies were counted the following day. For the bactericidal activities determined in tomato juice, each bacterial culture (100 µL) was added to 2 mL of PBS to measure the OD_600nm_. Each bacterial sample was further diluted to 1 × 10^3^ CFU of bacteria in 10 µL. Ten microliters (1 × 10^3^ CFU of bacteria) of the diluted bacterial culture was added to each of the 1.5-mL microcentrifuge tubes containing 1 mL of tomato juice, and they were incubated for 2 and 24 hours. The colonies were counted the following day.

### Molecular dynamics simulation

The input structures of tdAMP-1 and tdAMP-2 were predicted by Alphafold v2 (35). The model membrane-mimicking bacterial membrane was constructed using CHARMM-GUI with a CHARMM36 force field (58, 59). tdAMP-1, tdAMP-2, or Melittin (PDB: 6DST) was placed onto the model membrane and simulated using NAMD 3.0 (60) for 500 ns with three iterations. The model membrane was composed of PPPE (1-palmitoyl- 2-palmitoleoyl-phosphatidylethanolamine):PVPG (1-palmitoyl-2-vacenoyl-phosphatidylglycerol):PVCL2 (1,10-palmitoyl-2,20-vacenoyl cardiolipin)=16:4:1 (molar ratio). Note that the model membrane of tdAMP-2 contained a lipid concentration four times higher than that of tdAMP-1. This disparity is attributed to the larger spatial occupancy of tdAMP-2 compared to tdAMP-1, which is a result of its unique structural characteristics. The molar ratio of lipids remains consistent. The MD simulations were conducted using a 150-mM NaCl solution at a temperature of 310 K (equivalent to 37°C). The simulations were performed under constant particle number, pressure, and temperature (NPT) conditions. The water thickness was set to 20 Å using the TIP3P model. The system was neutralized using Na^+^ ions and subsequently equilibrated through a six-step process, which included energy minimization. Two femtoseconds per step were implemented for 500 nanoseconds of the production process. The analysis of hydrogen bonds formed between tdAMP-1 or tdAMP-2 and the model membrane was conducted using UCSF Chimera software (54).

### Live/dead staining

Bacteria were grown overnight in 2 mL of LB medium at 37°C, and 100 µL of bacterial culture was added to 2 mL of PBS to measure the OD_600nm_. Bacteria corresponding to 1 × 10^8^ CFU/mL of bacteria was collected from the diluted sample, re-suspended to 10^8^ CFU/mL of bacteria, and 10 µL (10^6^ CFU of bacteria in 10 µL) was added into 90 µL of PBS including 64 µg/mL of tdAMP-1, tdAMP-2, Melittin, or 90 µL of tomato juice. After incubating at 37°C for 45 minutes, the bacteria were stained with LIVE/DEAD BacLight Bacterial Viability Kit (Invitrogen, cat# L7012) for 15 minutes at 37°C. Ten microliters of sample was placed on a cover glass, imaged using a BZ-X810 (Keyence) microscope, and quantified with BZ-X810 image analyzer using Plan Fluorite 20X LD PH (BZ-PF20LP) objective.

### Statistical analysis

Data were tested for statistical significance with the GraphPad Prism software. The number of replicates for each experiment and the statistical test performed are indicated in the figure legends.

## Data Availability

The published article includes all datasets generated during this study.

## SUPPLEMENTAL MATERIAL

Figure S1 spectrum.03102-23-s0001.tif Figure S1, related to Fig. 1. *S*. Typhi grows well in both acidic and neutral pH conditions. Comparative growth assays on *S*. Typhi using media with pH levels of 4.5 and 7.4.

Figure S2 spectrum.03102-23-s0002.tif Figure S2, related to Fig. 2. The dialyzed tdAMP-1 and tdAMP-2 showed the same antimicrobial activities against *S*. Typhi. A–E, Growth curves and CFU assay results of *S*. Typhi treated with the dialyzed tdAMP-1 (A–B), tdAMP-2 (C–D), or acetate (E) at the indicated concentrations.

Supplemental material legends spectrum.03102-23-s0003.docx Legends of Fig. S1 and S2 and Table S1.

Table S1 spectrum.03102-23-s0004.xlsx Table S1, related to Fig. 2. Identification of tdAMPs. The genes encoding proteins with a length of less than 100 amino acids were analyzed using CAMPR3 and AMPpred, resulting in the top 20 candidates whose antibacterial probability exceeds 0.9. After careful evaluation of the candidates, we have identified the 4 candidates for this study: tdAMP-1 (YP_008563122.1), tdAMP-2 (XP_025885552.1), tdAMP-3 (XP_004247438.1), and tdAMP-4 (XP_004247441.1).

ASM does not own the copyrights to Supplemental Material that may be linked to, or accessed through, an article. The authors have granted ASM a non-exclusive, world-wide license to publish the Supplemental Material files. Please contact the corresponding author directly for reuse.
