# Supplementary figures and images for: Antimicrobial properties of tomato juice and peptides against typhoidal Salmonella
Source: Microbiol Spectr. 2024 Jan 30;12(3):e03102-23. doi: 10.1128/spectrum.03102-23 (PMC10913428; doi:10.1128/spectrum.03102-23)

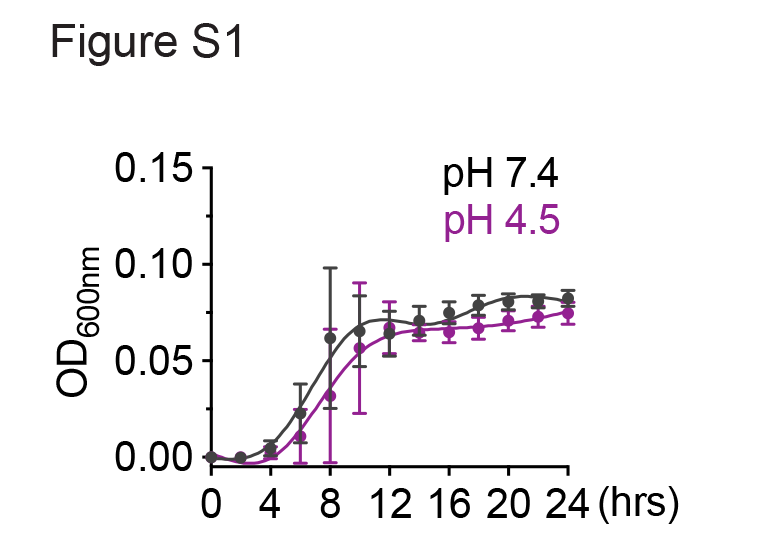

Supplement: Figure S1 — , related to Fig. 1. S. Typhi grows well in both acidic and neutral pH conditions. Comparative growth assays on S. Typhi using media with pH levels of 4.5 and 7.4. [file spectrum.03102-23-s0001.tif]

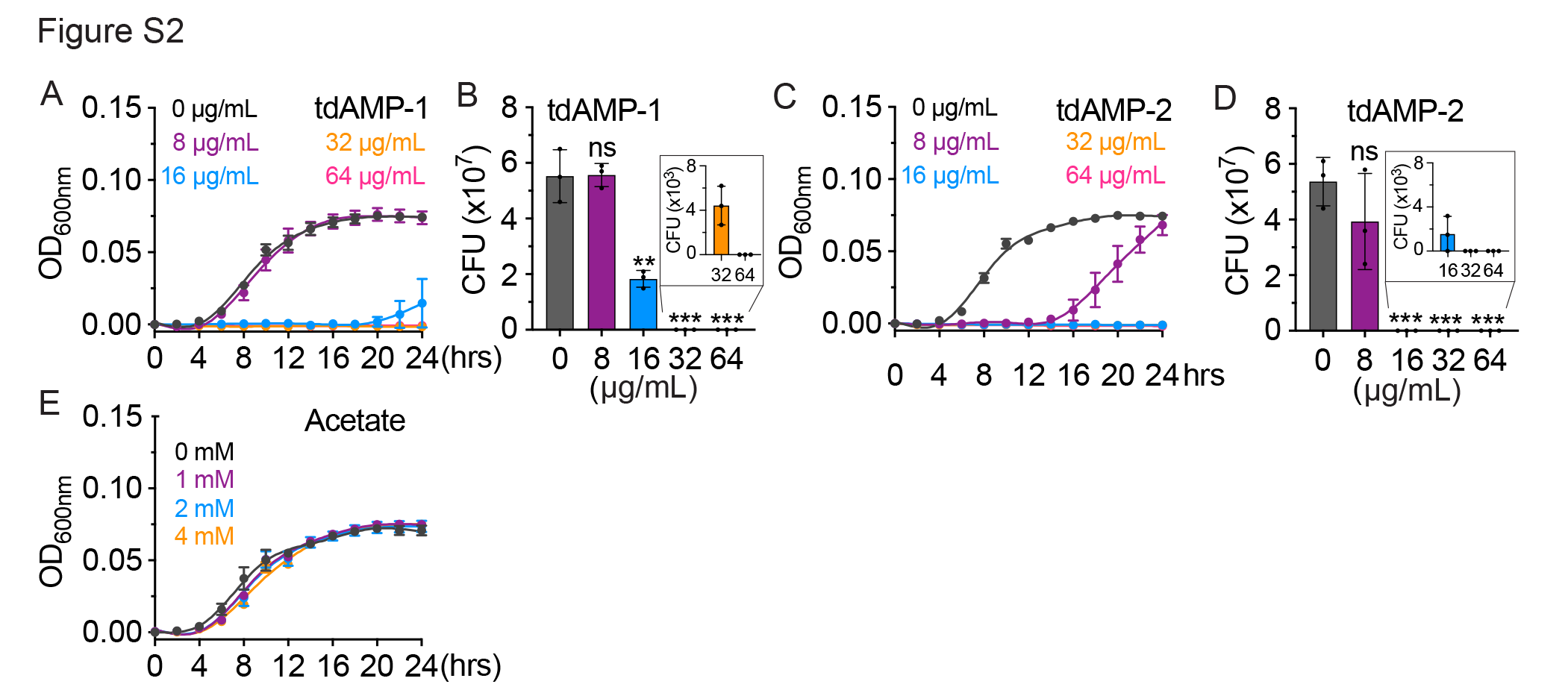

Supplement: Figure S2 — , related to Fig. 2. The dialyzed tdAMP-1 and tdAMP-2 showed the same antimicrobial activities against S. Typhi. A–E, Growth curves and CFU assay results of S. Typhi treated with the dialyzed tdAMP-1 (A–B), tdAMP-2 (C–D), or acetate (E) at the indicated concentrations. [file spectrum.03102-23-s0002.tif]
