# Supplementary material for: Antimicrobial properties of tomato juice and peptides against typhoidal Salmonella
Source: Microbiol Spectr. 2024 Jan 30;12(3):e03102-23. doi: 10.1128/spectrum.03102-23 (PMC10913428; doi:10.1128/spectrum.03102-23)
Supplement: Supplemental material legends — Legends of Fig. S1 and S2 and Table S1. [file spectrum.03102-23-s0003.docx]

**Figure S1, related to Fig. 1. *S.* Typhi grows well in both acidic and neutral pH conditions.** Comparative growth assays on *S.* Typhi using media with pH levels of 4.5 and 7.4.

**Figure S2, related to Fig. 2. The dialyzed tdAMP-1 and tdAMP-2 showed the same antimicrobial activities against *S.* Typhi.** **A-E**, Growth curves and CFU assay results of *S.* Typhi treated with the dialyzed tdAMP-1 (**A-B**), tdAMP-2 (**C-D**), or acetate (**E**) at the indicated concentrations.

**Table S1, related to Fig. 2. Identification of tdAMPs.** The genes encoding proteins with a length of less than 100 amino acids were analyzed using CAMPR3 and AMPpred, resulting in the top 20 candidates whose antibacterial probability exceeds 0.9. After careful evaluation of the candidates, we have identified the 4 candidates for this study: tdAMP-1 (YP_008563122.1), tdAMP-2 (XP_025885552.1), tdAMP-3 (XP_004247438.1), and tdAMP-4 (XP_004247441.1).
